# Comparing the reconstruction of regulatory pathways with distinct Bayesian networks inference methods

**DOI:** 10.1186/1471-2164-13-S5-S2

**Published:** 2012-10-19

**Authors:** Adriano V Werhli

**Affiliations:** 1 Centro de Ciências Computacionais - C3, Universidade Federal do Rio Grande - FURG, RS, Brazil

## Abstract

**Background:**

Inference of biological networks has become an important tool in Systems Biology. Nowadays it is becoming clearer that the complexity of organisms is more related with the organization of its components in networks rather than with the individual behaviour of the components. Among various approaches for inferring networks, Bayesian Networks are very attractive due to their probabilistic nature and flexibility to incorporate interventions and extra sources of information. Recently various attempts to infer networks with different Bayesian Networks approaches were pursued. The specific interest in this paper is to compare the performance of three different inference approaches: Bayesian Networks without any modification; Bayesian Networks modified to take into account specific interventions produced during data collection; and a probabilistic hierarchical model that allows the inclusion of extra knowledge in the inference of Bayesian Networks. The inference is performed in three different types of data: (i) synthetic data obtained from a Gaussian distribution, (ii) synthetic data simulated with Netbuilder and (iii) Real data obtained in flow cytometry experiments.

**Results:**

Bayesian Networks with interventions and Bayesian Networks with inclusion of extra knowledge outperform simple Bayesian Networks in all data sets when considering the reconstruction accuracy and taking the edge directions into account. In the Real data the increase in accuracy is also observed when not taking the edge directions into account.

**Conclusions:**

Although it comes with a small extra computational cost the use of more refined Bayesian network models is justified. Both the inclusion of extra knowledge and the use of interventions have outperformed the simple Bayesian network model in simulated and Real data sets. Also, if the source of extra knowledge used in the inference is not reliable the inferred network is not deteriorated. If the extra knowledge has a good agreement with the data there is no significant difference in using the Bayesian networks with interventions or Bayesian networks with the extra knowledge.

## Background

The rapid increase in the availability and diversity of molecular biology data has enabled many discoveries and advances in different fields related with systems biology. Many of these studies were based in a single biological entity or the union of several such entities. Nowadays the research community is realizing that the complexity of an organism is related with the network of single entities rather than with the individual biological entity. It is now clearer that the joint acting of several components through a network of interactions plays a pivotal role in determining the development and sustainability of an organism. Therefore, the study of biological networks is highly relevant. The problem is that these intricate biological networks are mainly unknown. Since we have at our disposal many different types of measurements taken from the components of these networks one interesting approach would be to try to reconstruct such networks.

In the last few years, several methods for the reconstruction of regulatory networks and biochemical pathways from data have been proposed. These methods were reviewed for example in [1,2].

Differential Equations are the most refined mathematical method to describe biophysical processes. They can describe, for example, the intra-cellular processes of transcription factor binding, diffusion, and RNA degradation; see, for instance, [3]. Such detailed descriptions of the dynamics are essential to an accurate understanding of regulatory networks but they require substantial prior knowledge about the system under investigation. For instance it is necessary to specify how the entities of the system relate with each other and all the parameters of the biochemical reactions. Although differential equations are the most accurate way of representing regulatory networks their use is limited by the necessity of substantial prior knowledge about the system they are representing. At the other extreme is the coarse grain approach of clustering which has been widely applied to the analysis of microarray gene expression data [2,4]. Clustering methods have very low computational costs to extract qualitative information about co-expression, but they are not powerful enough to provide the inference of the detailed structure of the underlying biochemical signalling pathways.

A promising compromise between these two extremes are Machine Learning methods that allow interactions between the nodes in the network to be represented in an abstract way - without the level of detail of the underlying pathways described by Differential Equation models - and to infer these interactions from data in a systems context, that is, distinguishing direct interactions from indirect interactions that are mediated by other nodes in the domain.

A non exhaustive list of methods used to infer the structure of networks from data includes: a system of Coupled Differential Equations [3], Graphical Gaussian Models [5,6], Relevance Networks [7] and Bayesian Networks [8-10]. See [11] for a comparison of some of the methods aforementioned. The main focus in this study is the systematic comparison amongst different BNs approaches. In all the approaches investigated in this paper a score-based inference scheme is followed. In this scheme a score is assigned to a particular model (network structure) given some observed data. The approaches investigated are: (i) a simple BN, (ii) a BN which benefits from interventions that are made in the system of interest during the measurement of the data and (iii) a probabilistic model which enables the use of extra knowledge in the inference. Hereafter we name BNs with interventions as BN-I and BNs with extra knowledge as BN-E.

## Results

### Evaluation criteria

Not all of the edge directions in a Bayesian network can always be inferred. This is due to the existence of equivalence classes of networks [12] which may lead to partially directed graphs. In view of the presence of directed and partially directed graphs, we apply two different criteria to assess the performance of the methods. In one of the approaches the information about the edge directions is completely discarded. Whenever two nodes are connected by a directed edge this edge is replaced by an undirected one. This approach is called the undirected graph evaluation (UGE). The other approach considers a predicted undirected edge as the superposition of two directed edges, pointing in opposite directions. This approach is called the directed graph evaluation (DGE). The result of the MCMC simulation is a sample of network structures which leads to a matrix of marginal posterior probabilities associated with the edges in a network. This defines a ranking of the edges. This ranking defines a receiver operator characteristics (ROC) curve, where the relative number of true-positive (TP) edges is plotted against the relative number of false-positive (FP) edges. Ideally we would compare the whole ROC curves but this is impractical. Therefore, we use the area under the ROC curve (AUC). The AUC summarizes the results for all the thresholds. A perfect predictor would produce an AUC value of 1.00. Conversely, a random predictor would produce an AUC value around 0.50. In general, bigger area values represent better predictors.

### Inference results

MCMC simulations are performed for all the approaches and data sets twice in order to check convergence. The convergence is verified by plotting the posterior probabilities of the edges from two different simulations initializations and checking if the results are similar. Note that this is a necessary but not a sufficient condition for convergence. All the MCMC simulations are executed with 5 × 10^5^ steps from which the first half were discarded as burn-in.

The extra knowledge used in conjunction with the real data in the BN-E approach was obtained from Kyoto Encyclopedia of Genes and Genomes (KEGG) pathways database [13-15] as described in [16]. For the synthetic data sets, obtained from a Gaussian distribution and from Netbuilder, we used two distinct sources of extra-knowledge, one completely correct $\left(B_{100}\right)$, and one half correct i.e. half of the entries are correct and the other half is wrong, $\left(B_{50}\right)$.

**Figure 1:**
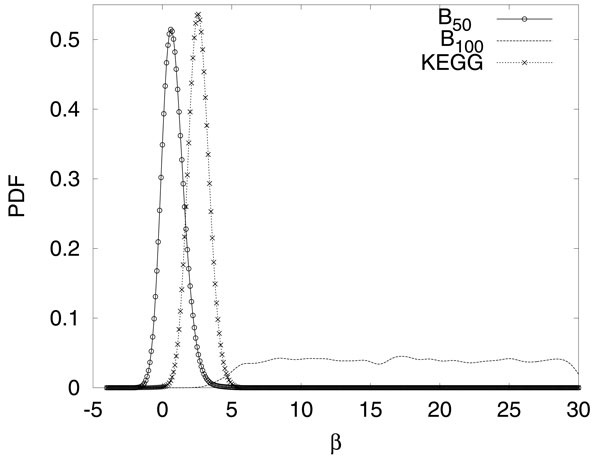
**Posterior distribution of hyper-parameter**. In the figure $B_{50}$ is the solid line with circles, $B_{100}$ is the dashed line and KEGG is the dotted line with crosses. This figure summarizes the typical hyper-parameter found by the BN-E for both sources of extra knowledge available, $B_{50}$ and $B_{100}$, when applied to the Gaussian and Netbuilder data and for the extra knowledge from KEGG applied to the Real data. The posterior distribution of the sampled hyper-parameters is estimated with a kernel estimator.

In Figure 1 we present a typical result of the hyper-parameters for the two different sources of extra-knowledge used with synthetic data, $B_{50}$ and $B_{100}$, and for Real data, KEGG. The results presented are the posterior distribution of the hyper-parameters obtained with a kernel estimator. In other words, the hyper-parameter is sampled with an MCMC scheme and in Figure 1 it is shown the distribution of these sampled hyper-parameters. The values of the hyper-parameter are an indicator of how much the extra knowledge agrees with the available data. The completely correct extra-knowledge, $B_{100}$, is fully integrated in the inference as is indicated by the wide range of large values sampled to the hyper-parameter. Conversely, when considering the half correct extra-knowledge, $B_{50}$ , the low values of the sampled hyper-parameter indicates that the extra-knowledge available is not completely integrated in the inference. For the Real data we can see that the extra knowledge from KEGG is not in complete agreement as is $B_{100}$ but it presents a slightly higher agreement in terms of the hyper-parameter than the one presented when considering $B_{50}$ .

**Figure 2:**
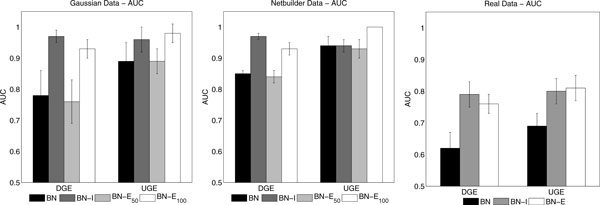
**Comparison of reconstruction accuracy**. Each sub-figure presents the results for one type of data set as indicated at the top of the sub-figure. For each data set type there are two groups of results, one obtained when taking the edge directions into account (DGE) and the other obtained when taking only the skeleton of the network into account. Within a figure each bar represents the AUC average over five data sets for different methods which are indicated in the legend of the sub-figures. The errorbars show the respective standard deviations. For Real data only one source of extra-knowledge is used, therefore, there is one less bar in the results.

In Figure 2 it is presented a comparison of reconstruction accuracy. Each of the three sub-figure presents the results for one type of data set, Gaussian, Netbuilder and Real data. For each data set type there are two groups of results, one obtained when taking the edge directions into account (DGE) and the other obtained when taking only the skeleton of the network into account. Each bar represents the AUC value, averaged over five data sets, for the different methods as indicated in sub-figures legend. The errorbar shows the respective standard deviations. For Real data only one source of extra-knowledge is used, therefore, there is one less bar in the results.

## Discussion

Figure 1 provides an indication about how the method BN-E can benefit from different types of extra knowledge. We can observe that when the method is associated with the extra knowledge, $B_{100}$, the extra knowledge is effectively used as is indicated by the wide distribution of the sampled hyper-parameters. Conversely, when the method is associated with extra knowledge that is not in perfect agreement with the data, as is the case in $B_{50}$ and KEGG, it moderately uses the extra knowledge as is evidenced by the distribution of their sampled hyper-parameters close to zero.

One interesting aspect observed is the behaviour of the hyper-parameter of the BN-E approach and the reconstruction accuracy obtained with this method. As we can see from Figure 1 the sampled hyper-parameter for the extra knowledge $B_{50}$ is much smaller than the one sampled for $B_{100}$. It is important to notice that this is the expected behaviour of the hyper-parameter. If we observe Figures 2 left and center we can see that the accuracy of the recovered structure is much higher for $B_{100}$ than for $B_{50}$ and that this difference is more pronounced for the DGE evaluation criterion. Also, note that the AUC values obtained for $B_{50}$ are in fact very similar to the ones obtained by the BN approach. This behaviour suggests that in order to the BN-E approach to take advantage from the extra knowledge available it has to have a very good agreement with the data. Moreover, if the data and the extra knowledge are not in agreement i.e. the extra knowledge is not beneficial to the inference, the BN-E approach does not suffer any impairment.

Observing the results for synthetic data in Figures 2 left and central we see that the methods BN-I and BN-E_100_ clearly outperform the others specially when considering the edge directions (DGE criterion). This suggests that the increase in the accuracy of the recovered networks is related with the edge directions i.e. these methods provide a way to the break up the symmetries which imply in the equivalence classes. It is also possible to note that the addition of the half correct extra knowledge, $B_{50}$, did not improve the results obtained with the BN approach. It is important to emphasize that the extra knowledge $B_{50}$ has half of its entries correct and the other half incorrect. As there is only one hyper-parameter associated with the extra knowledge if this hyper-parameter increases the inference method would use wrong information and certainly the result would be worse than without any extra knowledge. Therefore, this explains why the BN-E_50_ approach had the hyper-parameter sampled at very small values and, hence, has not improved the accuracy of the reconstructed network.

In Figure 2 right we see that both BN-I and BN-E outperformed BN increasing substantially the accuracy of the recovered networks for the Real data. Interestingly though, in this case both the DGE and UGE criteria have benefited of the extra information introduced by both approaches. We see that the hyper-parameter associated with the KEGG prior is slightly higher than the hyper-parameter associated with the $B_{50}$. This is explained by the construction of the KEGG prior where unknown entries were regarded as unknown $\left(B_{i,j}=0.5\right)$ as opposite to the extra knowledge $B_{50}$ where half of the entries are wrong.

## Conclusion

BNs are very attractive to the inference of the structure of networks by various reasons. One of the main advantages of BNs is its flexibility. In this paper we compared different BNs approaches where two of them are extensions of the classical BNs framework. The essence of both of these extensions of BNs is the inclusion of knowledge other than the data in the inference. If the BN-I interventions are taken into consideration and in the BN-E extra knowledge is added to the learning scheme.

Observing the results in Figure 2 we can conclude that both BN-I and BN-E_100_ perform better than the simple BN. This performance is significantly better when the comparison takes into account the edge directions (DGE score). This leads to the conclusion that both methods in fact perform better because they are able to destroy the equivalence classes symmetries. Another interesting conclusion is obtained when we observe Figure 1 in conjunction with Figure 2. It is clear that in order to the BN-E to benefit from the available extra-knowledge this has to have a very good agreement with the data as is the case with the $B_{100}$ extra knowledge. If this is not the case, the behaviour is similar to the one presented by the extra knowledge $B_{50}$ where the hyper-parameter is sampled at values close to zero effectively switching off the use of the extra knowledge, hence not improving the AUC scores.

Interestingly there are no significant differences when comparing the two best methods, BN-I and BN-E_100_, as can be observed in Figure 2. Both performed equally well in all the data sets used in this work. Therefore, there is no clear indication about which of these methods should be used. Nonetheless, it is clear that using any of them is a great advantage over the simple BN. The decision to chose among BN-E and BN-I will have to be made according to the system in study and the data available. For systems in which there are plenty of available extra knowledge this study suggests that it is not necessary to perform such expensive experiments with interventions. Conversely, if the system under scrutiny does not have extra knowledge available, it may be advisable to perform experiments with interventions. It is worth to note that even if there is plenty of extra knowledge available it is impossible to know beforehand if this will be in good agreement with the data. One indication about the quality of the extra knowledge can be obtained by the observation of the distribution of the sampled hyper-parameters as it is presented in Figure 1.

The main conclusion is that the use of more refined Bayesian network models significantly improves the results. Both more refined methods, BN-E and BN-I, performed equally well and, hence, their choice should be made according to the quality and availability of the data obtained from the system under investigation.

## Methods

### Bayesian Networks - BNs

Bayesian Networks (BNs) are a combination of probability theory and graph theory. A graphical structure , a family of conditional probability distributions  and their parameters **q**, fully specify a BN. The graphical structure  of a BN consists of a set of nodes and a set of directed edges. The nodes represent random variables, while the edges indicate conditional dependence relations. The family of conditional probability distributions  and their parameters **q **specify the functional form of the conditional probabilities associated with the edges, that is, they indicate the nature of the interactions between nodes and the intensity of these interactions. A BN is characterized by a simple and unique rule for expanding the joint probability in terms of simpler conditional probabilities. This follows the local Markov property: *A node is conditionally independent of its non descendants given its parents*. Due to this property, the structure  of a BN has necessarily to be a directed acyclic graph (DAG), that is, a network without any directed cycles. Let *X*_1_, *X*_2_, ..., *X_N_*be a set of random variables represented by the nodes *i *∈{1, ..., *N*} in the graph, define *π_i_*[*G*] to be the parents of node *X_i_*in graph *G*, and let ${X_{\pi }}_{{}_{i}}\left[G\right]$ represent the set of random variables associated with *π_i_*[*G*]. Then we can write the expansion for the joint probability as $P\left(X_{\text{1}},\;...\;,X_{N}\right)\;=\prod_{i=1}^{N}\text{P}\left(X_{i}|{X_{\pi }}_{{}_{i}}\left[G\right]\right)$.

The task of learning a BN structure in a score-based approach consists in devising a BN structure from a given set of training data . The main aim is to find a DAG structure that better explains the data available for learning. If we define that  is the space of all models, the first goal is to find a model $M^{*}\in M$ that is most supported by the data ,$M*=\;\text{arg}\underset{M}{\text{max}}\left\{P\left(M|D\right)\right\}$. Having the best structure $M^{*}$ and the data , we can now find the best parameters, $q=\text{arg}\underset{q}{\text{max}}\{P\left(q|M^{*},D|\right\}$ If we apply Bayes' rule we get P(M|D)∞P(D|M)P(M) where the marginal likelihood implies an integration over the whole parameter space:

(1) P(D|M) = ∫ P(D|q,M)P(q|M)dq

The integral in Equation 1, our score, is analytically tractable when the data is complete and the prior P(q|M) and the likelihood P(D|q,M) satisfies certain regularity conditions [17,18].

According to Equation 1 we have a way to assign a score to a graphical structure given a data set. However, the search for high scoring structures is not trivial. It is impossible to list the whole set of structures because its number increases super-exponentially with the number of nodes. Also when considering an sparse data set P(M|D) is diffuse, meaning that P(M|D) will not be properly represented by a single structure $M^{*}$. Hence, a Markov chain Monte Carlo (MCMC) scheme is adopted [19], which under fairly general regularity conditions is theoretically guaranteed to converge to the posterior distribution [20]. Given a network structure $M_{\text{old}}$, a network structure $M_{\text{new}}$ is proposed from the proposal distribution $Q\left(M_{\text{new}}|M_{\text{old}}\right)$, which is then accepted according to the standard Metropolis-Hastings [20] scheme with the following acceptance probability:

(2) $$A=\text{min}\left\{\frac{P\left(D|M_{\text{new}}\right)P\left(M_{\text{new}}\right)Q\left(M_{\text{old}}|M_{\text{new}}\right)}{P\left(D|M_{\text{old}}\right)P\left(M_{\text{old}}\right)Q\left(M_{\text{new}}\right)\left(M_{\text{old}}\right)},1\right\}$$

In this paper we use the standard MCMC proposal which consists in to propose, at each interaction, one of the basic operations of adding, removing or reversing an edge. For more details about this scheme see [10].

### Bayesian Networks with Interventions - BN-I

Nowadays molecular biology has different techniques for producing interventions in biological systems, for instance, knocking genes down with RNA interference or transposon mutagenesis. The consequence is that the components of the system which are targeted by the interventions are no longer subject to the internal dynamics of the system under investigation. The components of the biological system can be either activated (up-regulated) or inhibited (down-regulated) and under this external intervention their values are no longer stochastic. The intervened components are not subject to the internal dynamics of the system, hence their values are deterministic. However, the other components which are not intervened are influenced by these deterministic values. Therefore, interventions are very useful to break the symmetries within the equivalence classes of BNs and consequently to the discovery of putative causal relationships. For a discussion about equivalence classes see [21] and for a discussion about putative causal relationships see [12,22].

In order to incorporate the interventions under the BN framework two small modifications are necessary. The calculation of the score for observational data P(D|M) as defined in Equation (1) is modified. Effectively the measurements of a node *X_i_*under intervention are removed from the computation of the score.

The second necessary modification is related to the definition of equivalence classes. In [23] it is defined the Transition Sequence equivalent networks (TS-equivalent). Two networks $M_{1}$ and $M_{2}$ are TS-equivalent if and only if they have the same skeleton, the same set of v-structures and the same set of parents for all manipulated variables. All edges connected with an intervened node become directed when the concept of TS-equivalence is applied. Therefore, new v-structures are formed and further edges become directed. In order to obtain the TS-equivalent DAG the procedure presented by [24] is applied. For each intervened node in the network two dummy nodes are added each with one directed edge pointing from the dummy node to the intervened node. The new DAG now with the dummy nodes added is converted to a CPDAG (for a discussion about CPDAGs see [25] ). Finally the dummy nodes are removed and we have the DAG TS-equivalent graph.

The sampling scheme of the BNs-I is the same of the BNs and is given by Equation 2.

### Bayesian Networks with addition of Extra knowledge - BN-E

In order to be able to incorporate extra knowledge in the inference of networks it is necessary to define a function that measures the agreement between a given network structure  and the extra knowledge that we have at our disposal, . We call this agreement measure 'energy' following the approach proposed in [26].

A network structure  is represented by an adjacency matrix where each entry $M_{ij}$ can be either 0 or 1 representing respectively the absence and the presence of an edge between node-*i *and node-*j*. The prior knowledge matrix, or belief matrix, , is defined to have entries $B_{ij}$ ∈ [0, 1] representing our knowledge about the node interactions. An entry $B_{ij}=0.\text{5}$ denotes that we do not have any information about the presence or absence of an edge between node-*i *and node-*j*. If $0\leq B_{ij}<0.\text{5}$ we have prior evidence that the edge between node-*i *and node-*j *is absent and the evidence is stronger as $B_{ij}$ is closer to 0. At last, if $0.5<B_{ij}\leq 1$ we have prior evidence that there is a directed edge pointing from node-*i *to node-*j*. The evidence is stronger as $B_{ij}$ is closer to 1. It is important to note that the entries in our belief matrix are not proper probabilities and they only express our belief, or knowledge obtained from other sources, about the relationships among nodes.

Having defined how to represent a BN structure, , and the extra belief, , the energy of a network is defined as:

(3) $$E\left(M\right)=\overset{N}{\underset{i,j=1}{\sum }}|B_{i,j}-M_{i,j}|$$

where *N *is the total number of nodes in the studied domain. From Equation 3 it is clear that the energy *E *is zero for a perfect match between the prior knowledge  and the actual network structure , while increasing values of *E *indicate an increasing mismatch between  and .

Following the work of [26] we integrate the prior knowledge expressed by Equation 3 into the inference procedure, and define the prior distribution over network structures, , to take the form of a Gibbs distribution:

(4) $$P\left(M|\beta \right)=\frac{e^{-\beta E\left(M\right)}}{\underset{M\in M}{\sum }e^{-\beta E\left(M\right)}}$$

where the energy E() was defined in Equation 3, *β *is a hyper-parameter that corresponds to an inverse temperature in statistical physics, and the denominator is a normalizing constant that is usually referred to as the partition function. Note that the summation in the denominator extends over the set of all possible network structures. The hyper-parameter *β *can be interpreted as a factor that indicates the strength of the influence of the prior knowledge relative to the data. For *β *→ 0, the prior distribution defined in Equation 4 becomes flat and uninformative about the network structure. Conversely, for *β *→ ∞, the prior distribution becomes sharply peaked at the network structure with the lowest energy.

For Dynamic Bayesian Networks the summation in the denominator of Equation 4 can be computed exactly and efficiently as discussed in [16] with

(5) $$Z\left(\beta \right)=\underset{n}{\prod }\underset{\pi_{M}\left(n\right)}{\sum }e^{-\beta \varepsilon \left(n,\pi_{M}\left(n\right)\right)}.$$

In this paper we apply the method only to static BNs and thus the summation in the denominator of Equation 4 is in fact an upper bound to the true value. This happens because this summation includes all possible structures and we are only interested in the DAG structures. Furthermore, throughout this paper we use a fan-in restriction of three as has been proposed in several other applications, for instance see [27-29]. This fan-in restriction makes the summation over all structures closer to the summation of only the DAGs as it reduces the number of densely connected networks. The partition function approximation has been investigated elsewhere [16,30] and was not found to pose a problem to the proposed method.

### BN-E MCMC sampling scheme

At this point, having the prior probability distribution over network structures defined, an MCMC scheme to sample both the hyper-parameters and the network structures from the posterior distribution P(M,β|D) is proposed.

**Figure 3:**
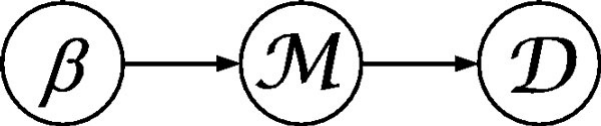
**Probabilistic Model**. The probabilistic graphical model represents conditional independence relationships between the data , the network structure , and the hyper-parameter of the prior on . The conditional independence relationships can be obtained from the graph according to the standard rules of factorization in Bayesian networks, as discussed, e.g., in [34]. This leads to the following expansion: P(D,M,β)=P(D|M)P(M|β)P(β).

A new network structure $M_{\text{new}}$ and a new hyper-parameter $\beta_{\text{new}}$ are proposed respectively from the proposal distributions $Q\left(M_{\text{new}}|M_{\text{old}}\right)$ and $R\left(\beta_{\text{new}}|\beta_{\text{old}}\right)$. This proposed move is then accepted according to the Metropolis-Hastings update rule [20] with the following acceptance probability:

(6) $$\begin{array}{c} A=\text{min}\left\{\frac{P\left(D|M_{\text{new}}\right)}{P\left(D|M_{\text{old}}\right)}\times \frac{P\left(M_{\text{new}}|\beta_{\text{new}}\right)}{P\left(M_{\text{old}}|\beta_{\text{old}}\right)}\times \frac{P\left(\beta_{\text{new}}\right)}{P\left(\beta_{\text{old}}\right)}\right)\times \\ \;\;\;\;\;\;\;\;\;\;\;\;\left(\frac{Q\left(M_{\text{old}}|M_{\text{new}}\right)}{Q\left(M_{\text{new}}|M_{\text{old}}\right)}\times \frac{R\left(\beta_{\text{old}}|\beta_{\text{new}}\right)}{R\left(\beta_{\text{new}}|\beta_{\text{old}}\right)},1\right\} \end{array}$$

which was expanded following the conditional independence relationships depicted in Figure 3.

In order to increase the acceptance probability which in turn can augment the mixing and convergence of the Markov chain the move is separeted into two submoves. In the first move a new network structure $M_{\text{new}}$ is sampled from the proposal distribution $Q\left(M_{\text{new}}|M_{\text{old}}\right)$ while keeping the hyper-parameter *β *fixed. Next, we sample a new hyper-parameter *β *from the proposal distribution $R\left(\beta_{\text{new}}|\beta_{\text{old}}\right)$ for a fixed network structure . The two sub-moves are iterated until a convergence criterion is satisfied.

### Data sets

**Figure 4:**
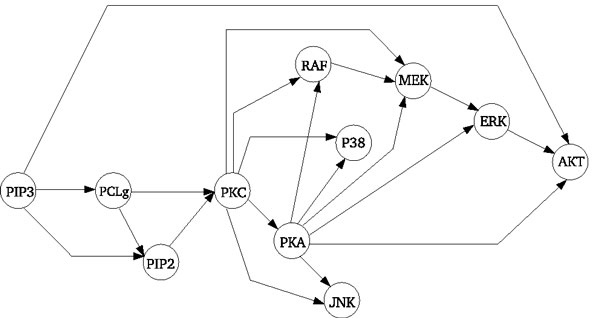
**Raf signalling pathway**. The graph shows the currently accepted signalling network, taken from [31]. Nodes represent proteins, edges represent interactions, and arrows indicate the direction of signal transduction.

One very interesting aspect when comparing different methods applied to the inference of the structure of networks is the ability to compare how they perform when faced with real data sets. In our case a real data set means data obtained with real experiments from a real biological system. Also, the comparison among the methods with real data is only possible if the network which the data was generated from is known. We call this known network the *gold-standard *network. Taking these considerations into account we use data from flow cytometry experiments obtained by [31] where the Raf signalling pathway, see Figure 4, was studied. This particular data set is very interesting as it provides high quality measurements, large amounts of data, intervened data and a *gold-standard *network.

Because the interest is to compare the BNs approaches in the context of inference of networks, where the data available are usually sparse, we down sampled the original data to 100 data points. Furthermore, we average the results over five data sets. The observational data is obtained from the original data where no interventions were realized. The interventional data is sampled from all the interventions realized in the original data and is composed by: 16 data points without intervention; 14 data points for each of the inhibited proteins (*AKT, PKC, PIP2, MEK*) and 14 data points for each of the activated proteins (*PKC, PKA*) proteins, performing a total of 100 data points.

In order to further investigate how the methods compare synthetic data sets were also prepared. These data are obtained from two different sources: a linear Gaussian distribution and a simulation tool named Netbuilder [32,33]. In both cases, the data is obtained from the known structure of Figure 4.

Considering (.) to denote the Normal distribution, a random variable *x_i_*is sampled from a linear Gaussian distribution with value distributed according to $x_{i}\sim N\left(\sum_{k}w_{ik}x_{k},\sigma^{2}\right)$ where the sum extends over all parents of node *i *and *xk *represents the value of node *k*. The standard deviation is set to *σ *= 0.1 and the interaction strengths |*w_ik_*| are sampled from the uniform distribution over the interval [0.5, 2], where the sign of *w_ik_*is randomly varied.

In Netbuilder a sigma-pi formalism is implemented as an approximation to the solution of a set of Ordinary Differential Equations that models enzyme-substrate reactions, allowing the acquisition of data typical of signals measured in molecular biology. The data sets simulated with Netbuilder are closely related to real data sets when compared with the Gaussian data. For more details about the data generation see [11].

## Competing interests

The authors declare that they have no competing interests.
